# ING Genes Work as Tumor Suppressor Genes in the Carcinogenesis of Head and Neck Squamous Cell Carcinoma

**DOI:** 10.1155/2011/963614

**Published:** 2010-10-28

**Authors:** Xiaohan Li, Keiji Kikuchi, Yasuo Takano

**Affiliations:** ^1^Kanagawa Cancer Center Research Institute, 1-1-2 Nakao, Asahi-ku, Yokohama 241-0815, Japan; ^2^Division of Pathology, Affiliated Shengjing Hospital of China Medical University, Shenyang 110004, China

## Abstract

Head and neck squamous cell carcinoma (HNSCC) is the sixth most common cancer in the world. The evolution and progression of HNSCC are considered to result from multiple stepwise alterations of cellular and molecular pathways in squamous epithelium. Recently, inhibitor of growth gene (*ING*) family consisting of five genes, *ING1* to *ING5*, was identified as a new tumor suppressor gene family that was implicated in the downregulation of cell cycle and chromatin remodeling. In contrast, it has been shown that *ING1* and *ING2* play an oncogenic role in some cancers, this situation being similar to TGF-*β*. In HNSCC, the *ING* family has been reported to be downregulated, and ING translocation from the nucleus to the cytoplasm may be a critical event for carcinogenesis. In this paper, we describe our recent results and briefly summarize current knowledge regarding the biologic functions of *ING* in HNSCC.

## 1. Introduction

Head and neck squamous cell carcinoma (HNSCC) is the sixth most common cancer in the world. More than 500,000 new cases and the over 50% mortality rate annually indicate a major health problem worldwide [1]. HNSCC is a broad term that represents squamous cell carcinomas that arise in the upper aero- and digestive tract, including the larynx, the pharynx, and the oral cavity. These sites form a functional and anatomic unit and share exposure to the same etiological factors in carcinogenesis [1]. It is well known that smoking and alcohol abuse are major risk factors for HNSCC. Additionally, human papillomavirus (HPV) infection is another implicated risk factor, in particular for oropharyngeal SCC [2, 3]. 

The evolution and progression of HNSCC are considered to result from multiple stepwise alterations of cellular and molecular pathways in the squamous epithelium [4]. Although lifestyle factors account for the majority of HNSCCs, genetic alterations will cause some individuals to be more sensitive to these environmental factors. Therefore, screening for reliable genetic changes can provide a possible opportunity to predict the risk of malignant transformation. Tumor suppressor genes (TSGs) are often referred to as “gatekeepers” because they prevent cancer development by direct control of cell growth through genes such as *p53* and* p16, *the inactivation of which has been reported in many tumors. The alterations of TSG, including mutation, loss of heterozygosity (LOH), and microsatellite instability, are considered to increase genetic susceptibility for malignant transformation. Previous studies have identified that alterations of* p53* and *p16* are associated with the development and progression of HNSCC [5–8]. Inhibitor of growth gene (*ING*) family, a new candidate TSG class, is implicated in cell cycle control, senescence, apoptosis, DNA repair, and chromatin modeling. The loss or downregulation of* ING* expression has been observed in HNSCC. In this paper, we summarized current knowledge on the biological function of* ING *family members and their status in the tumorigenesis of HNSCC.

## 2. ING Gene Family

*ING1*, the first member of the *ING* family, was discovered through a subtractive hybridization assay between normal mammary epithelium and breast cancer cell lines and was shown to play a role in neoplastic transformation [9]. Subsequently, four other members of *ING* family,* ING2*,* ING3*,* ING4*, and *ING5*, were identified by computer homology searches and were shown to have 32 to 76% DNA sequence homology with *ING1 *[10–13]. The* ING* genes each mapped to independent chromosomes: 13q34, 4q35, 7q31, 12p13.3, and 2q37.3. All of the* ING* genes except *ING3* localize to the subtelomeric region of their respective chromosomes [14]. In addition, phylogenetic analysis identified that *ING* genes are conserved in many species, including humans, mice, rats, and yeast [15]. Alignment data show that the human and mouse ING1 and ING3 proteins are 90% identical, whereas the human and frog ING1 and ING3 proteins are 81% and 82% identical, respectively [16]. These data suggest that *ING* genes play important roles in biological processes central to life.

Most *ING* genes, excluding *ING5*, encode variants due to different promoters, exons, and alternative splice variants. ING1 encodes four isoforms, p47^ING1a^, p33^ING1b^, p24^ING1c^, and p27^ING1d^, which vary in mass between 24 and 47 kDa. Among these isoforms, p33^ING1b^ is the most widely expressed in normal tissues [17]. ING2 encodes two isoforms. ING2a, also called ING2 and ING1L, encodes a 280-aa protein (p33^ING2^) that shares 58.9% similarity with p33^ING1b^ [10, 11]. Recently, ING2b was identified and shown to be transcribed from the middle of intron 1 of ING2a [18]. In addition, ING3 encodes two isoforms, p47^ING3^ and p11^ING3^ [12]. ING4 encodes eight splice variants: ING4_v1, v2, v3, v4, ING4ΔEx2, ΔEx3, ΔEx6A, and ΔEx6B [19, 20]. Only ING5 encodes a unique 240-aa protein (p28^ING5^) [13]. Splice variants of ING proteins may compensate or compete with each other and create more diversity in ING functions.

## 3. The Structure and Function of ING Proteins

**Figure 1:**
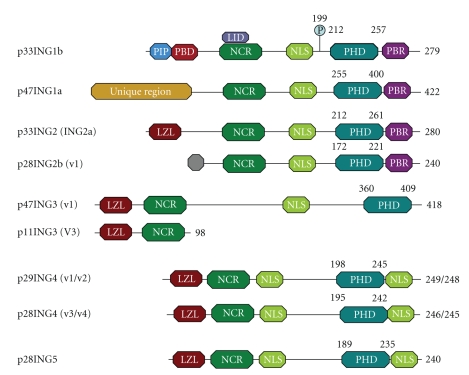
Structure of ING proteins in *Homo sapiens*. Each ING protein with its name and encoding major variants is listed on the left. The characterized domain composition, approximate location, is shown on the right. All ING proteins contain three conserved regions, a PHD (plant homeodomain), NLS (nuclear localization signal), and NCR (novel conserved region) from C-terminal region to N-terminal region. An LZL (leucine zipper-like domain) is present in ING2-5. p33^ING1b^ also have a PIP (PCNA-Interacting Protein Motif) domain through which it binds to PCNA following UV irradiation, a PBD (partial bromodomain) which commonly found in chromatin-associated protein, and an LID (Lamin Interaction domain). p33^ING1b^ binds to lamin A/HDAC complexes via LID to maintain its levels and biological function in nucleus. Additionally, phosphorylation sites were found at serine 199 of p33^ING1b^. 14-3-3 bind to phosphorylated serine 199 result in translocation of p33^ING1b^ from the nucleus to the cytoplasm.

All ING proteins contain a plant homeodomain (PHD) in the C-terminal region, a nuclear localization signal (NLS), and a domain with an unknown function called the novel conserved region (NCR) (Figure 1). The N-terminal region of each ING protein is unique, which determines the differential structures of ING proteins [21]. The PHD domain, a zinc finger domain that binds histone H3 in a methylation-sensitive manner, has been implicated in chromatin remodeling [22]. Localization of ING proteins in the nucleus is critical to their function [23]. The NLS targets ING1 or other ING proteins to different chromatin domains in the nucleus and nucleolus in response to UV-induced DNA damage [24]. Moreover, a leucine zipper-like (LZL) domain is present in ING2–5 and has the potential to form a hydrophobic face near the N-terminus of ING proteins [25]. Regarding its function, the LZL domain may be linked to nucleotide excision repair and induction of apoptosis [26]. p33^ING1b^ also carries three other domains. A proliferating cell nuclear antigen-(PCNA-) interacting protein motif (PIP) domain binds with PCNA following UV irradiation. A partial bromodomain (PBD) is commonly found in chromatin-associated protein. A lamin interaction domain (LID) binds with lamin A/HDAC complexes to maintain its levels and biological function in the nucleus [27]. Recently, a phosphorylation site was found at serine 199 of p33^ING1b^. 14-3-3 family proteins can bind to phosphorylated serine 199, resulting in translocation of p33^ING1b^ from the nucleus to the cytoplasm [28].

## 4. PHD Domain and Epigenetic Control

Although the* ING1* gene was cloned as a candidate gene for tumor suppression, studies on the effects of overexpression or downregulation of ING family proteins on various cellular processes imply that the roles of the *ING* family genes in tumorigenesis depend on cellular contexts; they could also function as oncogenes in several aspects [29]. Therefore, we first described the functions of ING family proteins in the epigenetic control of gene transcription and DNA replication, details of which are now going to be elucidated.

Epigenetic control of gene transcription is attained partly by modulation of covalent modifications such as acetylation, methylation, and/or phosphorylation of nucleosomal histones within gene promoters [30]. ING proteins are known to be a component of either histone acetylase (HAT) complexes or histone deacetylase (HDAC) complexes that activate and inactivate gene transcription, respectively. p33^ING1b^ interacts with the mSin3/HDAC complex and also with proteins associated with HAT activity such as p300, inducing hyperacetylation of histones H3 and H4 [31–33]. Similarly, ING2 complex with p300 also serves as a component of the mSin3/HDAC complex [34–36]. ING3 associates with the hNuA4/Tip60 HAT complex (nucleosome acetyltransferase of H4 and Tat interactive protein, respectively; Tip60 is the human homolog of yeast Esa1 HAT) that is responsible for acetylation of histone H4 and H2A [36, 37]. Both ING4 and ING5 bind to p300 [13], but they also associate with different HAT complexes. ING4 is identified as a component of a four-subunit HAT complex containing HBO1 (histone acetyltransferase binding to origin recognition complex-1). HAT and its cofactors JADE1/2/3 preferentially acetylate histone H4 [36]. ING5 associates with MOZ (monocytic leukemic zinc-finger protein)/MORF (MOZ related factor) HAT and its cofactor BRPF (bromodomain-PHD finger protein) 1/2/3, resulting in increased specificity for acetylation of histone H3 lysine 14 [36].

In turn, the PHD domains of ING family proteins were recently shown to recognize trimethylated lysine 4 of histone H3 (H3K4me3) that in many cases associates with active gene transcription [30]. ING2 was firstly shown to bind H3K4me3 via its PHD domain and stabilize the mSin3-HDAC complex, resulting in repression of DNA damage-induced transcription of cyclin D1 gene [22, 38]. ING1 also binds H3K4me3, and this binding is somehow necessary for ING1-mediated DNA repair upon UV irradiation as well as doxorubicin-mediated induction of apoptosis in HT1080 fibrosarcoma cells [39]. Intriguingly, cancer-associated mutations in the ING1 PHD domain impaired the binding of ING1 to H3K4me3 with concomitant loss of functions in DNA repair and apoptosis, implying that the binding of ING1 to H3K4me3 underlies its tumor-suppressive functions [39]. Binding of ING4 to H3K4me3 and its biological outcomes were extensively studied [40–42]. Promoters bound by ING4 in response to DNA damage were identified using a chromatin immunoprecipitation technique followed by whole genome promoter tiling arrays [42]. ING4 was recruited to its target promoters upon interaction with H3K4me3 and increased the acetylation of histone H3 lysine 9, leading to activation of gene transcription and sensitization to cell death or inhibition of anchorage-independent cell growth [42].

These compiled lines of evidence indicate that one basic function of ING family proteins is to translate H3K4me3 markings on the nucleosomes into activation or inactivation of gene transcription, DNA replication, or DNA repair through the associated HAT or HDAC complex. Based on this simple framework, further questions as follows may be posed for the elucidation of the substance of “cellular contexts” as described above, the same family member of ING can complex with either HAT or HDAC. (1) What determines the combination of ING proteins and HAT or HDAC complexes and the final outcomes? (2) What modulates the inducible or constitutive binding of ING proteins to H3K4me3 within gene promoters? (3) Do ING family members compete for the binding to the same H3K4me3 within a promoter or have some specificity for it? To answer these questions, identification of the genes modulated by ING family proteins and side-by-side analyses of transcriptional response of the gene and factors that associate with ING proteins as made in [42] may be helpful.

## 5. ING and DNA Repair

The balance between cell growth and cell death is characterized in tissue development and homeostasis. In response to slightly stressful stimuli, cells usually start a cellular stress response including DNA repair to ensure survival. However, when irreversible damage accumulates, cells can permanently arrest the cell cycle (cellular senescence) or trigger a cell death program (apoptosis) [43]. 

*ING1* is the founding member of the ING family and the most well studied. Paul et al. confirmed that ING1 interacts specifically with three proteins, p38MAPK, mammalian JNK/p38MAP kinase (MEKK4), and RAD50, by utilizing a cross-species (yeast, fly, and human) bioinformatics-based approach. Both p38MAPK and MEKK4 participate in a well-defined stress response pathway. These novel ING-interacting proteins further link ING proteins to cellular stress and DNA damage signaling [44]. Nucleotide excision repair (NER) is a crucial stress response mechanism for maintaining genomic stability. Overexpression of p33^ING1b^ can enhance NER of both UV-damaged genomic DNA and exogenous plasmid DNA in a host-cell-reactivation assay. Moreover, p33^ING1b^ requires the participation of functional p53 in DNA repair and may be a crucial component in the GADD45-mediated NER pathway [45]. Conversely, missense mutations in the SAP30-interacting domain and PHD finger motif of *ING1* abrogated the enhancement of NER in a host-cell-reactivation assay and a radioimmunoassay [46]. In addition, PCNA is an essential processivity factor for DNA polymerases and functions in both eukaryotic chromosomal DNA replication and NER. p33^ING1b^ contains a PIP motif within its N-terminus. By competitively binding PCNA through its PIP domain, p33^ING1b^ may contribute to regulating the switch from DNA replication to DNA repair [47]. In both normal human epithelial keratinocytes (NHEKs) and a keratinocyte cell line, HaCaT, the expression levels of p33^ING1b^ were elevated by UV induction independent of p53 status, thus suggesting that ING1 may participate in the cellular stress response and skin carcinogenesis [48]. In addition, ING2 interacted with certain HAT or HDAC proteins through its LZL domain, instead of the PHD region, to regulate histone H4 acetylation, chromatin decondensation, and NER [35]. Therefore, ING proteins may participate in DNA repair through the regulation of the NER pathway in response to cellular stress and DNA damage.

## 6. ING Proteins and Cell Cycle

Loss of proper control of the cell cycle is a major cause of cell transformation. Cellular senescence refers to the arrest in the G0 phase of the cell cycle [49]. p33^ING1b^ was upregulated in senescent human fibroblasts, and antisense p33^ING1b^ extends the proliferative lifespan of normal human fibroblasts [50]. Moreover, ectopic expression of ING1 in diploid human fibroblasts resulted in cell cycle arrest with some features of cellular senescence [51]. Chromatin immunoprecipitation analysis indicated that the chromatin binding affinity of p33^ING1b^ was higher in senescent cells compared with young cells, thus suggesting that ING1-mediated functions may be subject to age-dependent mechanisms of control directed to prevent induction of apoptosis in senescent but not in young cells [52]. ING2 enhanced the interaction between p53 and p300 and acted as a cofactor for p300-mediated p53 acetylation. Overexpression of ING2 induced senescence in young fibroblasts in a p53-dependent manner. Conversely, the downregulation of ING2 expression by siRNA transfection led to delaying the onset of senescence [53].

Previous research has demonstrated that overexpression of p33^ING1b^ increased the number of human diploid fibroblasts in the G0/G1 phase. Conversely, antisense p33^ING1b^ permitted these cells to enter S phase [9]. Cyclin E is a member of the cyclin family and binds to Cdk2 in the G1 phase, which is required for the transition from G1 to S phase. Expression of p33^ING1b^ in human hepatocellular carcinoma (HCC) was inversely correlated with cyclin E kinase activity by autoradiography [54], thus implicating that the reduction of p33^ING1b^ expression may contribute to the process of malignant transformation of HCC via an increase of cyclin E kinase activity. Another study indicated that ectopic expression of ING1b in H1299 cells sensitized the cells to short-term G2/M cell cycle delay [55]. In addition, adenovirus-mediated overexpression of ING1 in mouse mammary epithelial cells resulted in the downregulation of cyclin B1, which accumulates during the G2-M phase of the cell cycle [55]. Moreover, adenovirus-ING4-mediated transfection of PANC-1 human pancreatic carcinoma cells inhibited cell growth, altered the cell cycle with S-phase reduction and G2/M phase arrest, and induced apoptosis [56]. These findings suggest that ING may regulate cell senescence and cell cycle via the G1/S and the G2/M cell cycle checkpoints.

## 7. ING and Apoptosis

Apoptosis plays important roles in normal development and removal of the cells carrying severe DNA-damages induced by DNA damaging agents. In cancer cells, activation of pathways that favor cell survival instead of apoptosis may contribute to tumorigenesis. Many different agents and growth environmental factors can be used to induce apoptosis, such as cytotoxic drugs, irradiation, and serum starvation. Expression of ING1 increased upon the induction of apoptosis in P19 mouse teratocarcinoma cells by serum deprivation. Elevated expression of ING1 cooperated with c-myc gene expression to enhance the extent of apoptosis in P19 and rodent fibroblast cells [57]. Ectopic expression of p33^ING1b^ also sensitized cells to apoptosis induced by etoposide, taxol, and doxorubicin [24, 52, 55]. Ectopic expression of p33^ING1b^, but not p47^ING1a^, significantly enhanced UV- or hydrogen peroxide-induced apoptosis in young (low passage) but not senescent Hs68 cells. Moreover, cotransfection of p33^ING1b^ and p53 increased the percentage of apoptotic cells compared to transfection of either of these two proteins alone [52]. Conversely, expression of p33^ING1b^ antisense constructs protects cells against apoptosis [57] and promotes neoplastic transformation [9]. p33^ING1b^ activates transcription of the p21/WAF1 promoter, a key mechanism required for p53-mediated cell growth control [58]. Adenovirus-mediated transfer of p33^ING1b^ with p53 suggested an additive or synergistic effect on apoptosis in immortal human cancer cells [59]. In addition, p33^ING1b^ was demonstrated to influence tumor necrosis factor (TNF)-*α*-mediated apoptosis in Hs68 cell by upregulation of HSP70 expression and enhancement of the ability of TNF-*α* [60]. All ING proteins tested to date show the ability to regulate apoptosis in varying degrees through similar or different signal pathways. For example, the ING2 PHD finger interacts with phosphatidylinositol 5-phosphate (PtdIns5P) in vivo, and their interaction regulates the ability of ING2 to activate p53 and p53-dependent apoptotic pathways [61]. Increased ING2 expression was also found to increase Bax expression and enhance UVB-induced apoptosis in human melanoma cells [62]. Additionally, overexpression of ING3 significantly promoted UV-induced apoptosis through the activation of the Fas/caspase-8 pathway, and knockdown of ING3 remarkably decreased UV-induced apoptosis [63]. These results suggested that ING might induce apoptosis through varied pathways in response to different agents.

## 8. ING and p53

*p53 *is an important TSG that is inactivated in many cancers. p53 assimilates disparate input signals, including oncogene activation, DNA damage, mitotic impairment, and oxidative stress, to initiate appropriate outputs such as initiation of DNA repair, cell cycle arrest, senescence, or apoptosis [64]. The physical and functional interactions between ING and p53 have been investigated widely, but the conclusions are not consistent. In overexpression experiments, all ING proteins except ING3 have been observed to coimmunoprecipate with p53. Moreover, ING-induced cell cycle arrest and apoptosis were compromised in p53-deficient cultured cells [25, 65, 66]. Functional p53 is required for p33^ING1b^-mediated inhibition of cell growth in cultured cells. Furthermore, p33^ING1b^ was proposed to compete with murine double minute 2 (MDM2), an important negative regulator of p53, for the same binding site on p53, leading to an increase in the stability and activity of p53 [67]. ING2 may modulate p53-dependent chromatin remodeling, apoptosis, and DNA repair by functioning as a scaffold protein to mediate the interaction between p53 and p300 [35]. Additionally, overexpression of ING4 or ING5 leads to a reduction in colony-forming efficiency, inhibition of S-phase, and induction of apoptosis in a p53-dependent manner. ING4 and ING5 may stabilize p53 and enhance p53-mediated cellular responses to genotoxic stresses and apoptotic stimuli through ING4/5-mediated acetylation of p53 [13]. These results implicated that ING proteins may be significant modulators of p53 function. However, it was worth noting that the experiments of *ING1 *knockout mice and knockout cells indicated that ING1 functions were mostly independent from the p53 signaling pathway in physiological conditions [68, 69]. In mice, the *Ing1* gene decodes three spliced isoforms. Ing1a and Ing1c encode a 31 kDa protein, and Inglb encodes a 37 kDa protein (p33^ING1b^ in human). Loss of p37^Ing1^ induced BAX expression and increased DNA damage-induced apoptosis in primary cells and mice irrespective of p53 status. Moreover, p53 functions are unperturbed in p37^Ing1^-deficient cells. Moreover, p37^Ing1^ suppressed the formation of spontaneous follicular B-cell lymphomas in mice. Therefore, p37^Ing1^ can negatively regulate cell growth, apoptosis, and tumorigenesis in a p53-independent manner [69]. Previous studies also demonstrated that the expression of ING might be independent on p53 status in some tumor tissues. Decreased ING1 expression may play important roles in tumorigenesis of the specimens with expression of the wild-type p53 gene in gastric carcinoma [70] and nonsmall cell lung carcinoma (NSCLC) [71]. 

In addition, ING proteins are found to function in a p53-independent manner. One major p53-independent function of ING proteins may be negative regulation of NF-*κ*B. p33^ING1b^ and ING2 proteins were found to suppress expression of NF-*κ*B by upregulating HSP70 gene expression and augment TNF-*α*-induced apoptosis [60]. ING4 is shown to directly interact with p65(RelA) in glioma cells to inhibit transcriptional activity of NF-*κ*B. Correspondingly, the expression of NF-*κ*B-responsive genes is shown to be significantly increased in ING4 knockdown cells [72]. Another study showed that ING4 suppresses NF-*κ*B-regulated promoters by binding with both of p65 and H3K4me3 on the promoter [73]. This recruitment of ING4 accompanies the reduction of p65 phosphorylation and concomitant change of complex formation of p65 with p300 (HAT) to HDAC1 resulting in the decrease of acetylated histones and H3K4me3 within the promoter [73]. Additionally, ING4 was found to affect the stability of hypoxia inducible factor (HIF) and mediate HIF activity [72]. 

Based on these findings, ING and p53 may function independently in apoptosis pathways, but they can influence the activity of each other in tumorigenesis [15]. As epigenetic regulators of chromatin structure, ING proteins may amplify the effects of p53 on gene expression and also directly affect DNA repair and apoptosis independently of p53 by altering chromatin structure.

## 9. ING Genes and Tumorigenesis

**Figure 2:**
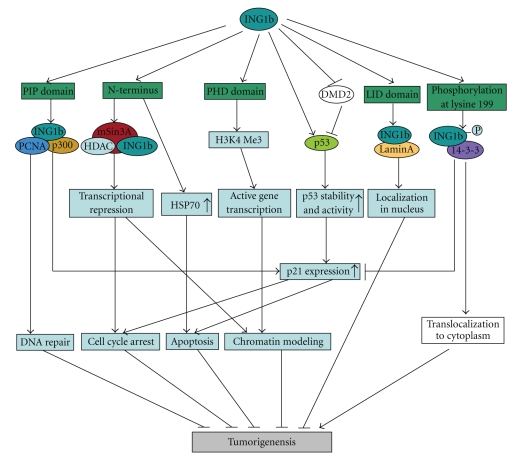
The role of p33^ING1b^ protein in tumor supression. p33^ING1b^ could recognize trimethylated lysine 4 of histone H3 (H3K4me3) by PHD domain and has been implicated in chromatin remodeling and activation of some genes transcription. This binding is somehow necessary for induction of DNA repair and cell death. p33^ING1b^ also associates with the Sin3/HDAC-mediated transcriptional repression through its unique N-terminal sequence and may be involved in repression of some essential cell cycle regulator genes. Moreover, p33^ING1b^ binds PCNA and p300 complex to promote DNA repair through a PIP motif in response to UV-irradiation and, subsequently, may trigger apoptosis by the induction of p21 expression. p33^ING1b^ competes with murine double minute 2 (MDM2) leading to an increase in the stability and activity of p53. p21, the one of the targets of p53, is also upregulated to involve in cell cycle arrest and the induction of apoptosis. Additionally, p33^ING1b^ could upregulate expression of HSP70 gene to induce apoptosis independently of p53 status. Furthermore, p33^ING1b^ binds to lamin A via LID domain to stabilize its level and biological function in nucleus. Conversely, 14-3-3 can bind to p33^ING1b^ with phosphorylated serine 199 and results in translocation of p33^ING1b^ from the nucleus to the cytoplasm, which may involve in tumorigenesis.

Previous studies have implicated members of the* ING* family as candidate type II TSGs that are involved in a variety of processes, including DNA repair, cell cycle control, senescence, apoptosis, and chromatin remodeling, which are critical points for genomic integrity and stability (Figure 2). Thus, loss or decrease of ING expression may be a potential key point in tumorigenesis. Knockout experiments demonstrated that ing1-dificient mice were more sensitive to total body gamma radiation, and loss of ing1 was associated with earlier onset and higher incidence of lymphomas [68].

Loss of nuclear p33^ING1b^ was observed in melanoma, seminoma, papillary thyroid carcinoma, ductal breast carcinoma, and acute lymphoblastic leukemia by comparing these neoplastic tissues with normal cells and tissues [74]. Until now, inactivation and reduced expression of *ING* genes has been reported in cancers of lung [71], breast [75], stomach [70, 76], esophagus [77], blood [78], brain [79], and HNSCC [80]. Interestingly, ING gene mutation is uncommon in cancer. In fact, translocation of ING proteins from the nucleus to the cytoplasm has been observed in some types of cancer, such as the tumors of the breast [75] and brain [79], melanoma [74], and lymphoblastic leukemia [78]. Therefore, the ING cellular compartment shift from the nucleus to the cytoplasm may cause loss of normal cellular function and may play a central role in tumorigenesis and progression.

Like other ING genes, nonphysiological overexpression of ING2 induces apoptosis and cell cycle arrest via p53 modification [10], and decreased ING2 expression was found in cutaneous cancer [81] and HNSCC [82]. However, expression of ING2 was upregulated in colorectal cancer [83], Burkitt's lymphoma, and cervical cancer [29]. Moreover, ING2 may bind to the RPB1-mSin3A-HDAC complex on the *MMP13* promoter to upregulate MMP-13 expression [83]. Thus, the function of ING2 may be different depending on the cancer type. A recent study suggested that ING2 is a novel mediator of transforming growth factor (TGF)-*β*-dependent responses in epithelial cells [84]. TGF-*β* is considered to have tumor suppressor-like functions in normal epithelium and also have oncogenic functions in invasive metastatic cancers. Therefore, ING2 may play different roles in normal cells and cancers by mediating the TGF-*β* signaling pathway.

## 10. Expression of ING Genes in HNSCC

**Figure 3:**
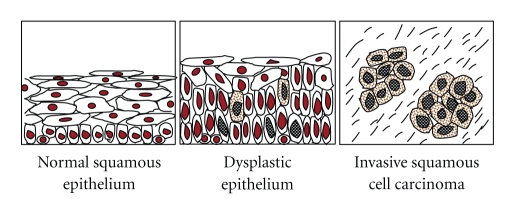
The schematic diagram of ING proteins expression in the malignant development of HNSCC. Nuclear expression of ING proteins is downregulated from normal squamous epithelium to dysplastic epithelium and invasive HNSCC. In contrast, the cytoplasmic expression of ING proteins in dysplastic epithelium and invasive HNSCC is gradually increased compared with normal squamous epithelium. The positive expression of ING proteins is shown with brown color.

Previous studies have demonstrated 45.5%–68% LOH of* ING *genes in HNSCC (Table 1), and 50%–76% decreases in the mRNA levels of *ING3–5*. In recent studies, we also investigated expression as well as the subcellular localization of ING proteins in 214 cases of HNSCC by immunohistochemistry. Decreased expression of p33^ING1b^, ING4, and ING5 in nuclei was observed in 36.9%, 61.3%, and 36% of the HNSCC cases, respectively. These results suggest that the loss or downregulation of nuclear expression of ING proteins participates in tumorigenesis of HNSCC. By contrast, mutations of the *ING* genes are rare (0–4.3%) in HNSCC although most of the mutations are present in the domains critical for the functions of ING proteins (Table 1), suggesting that mutations are not the major cause for *ING* family inactivation. In addition, the shift of p33^ING1b^ from the nucleus to the cytoplasm was observed in 24.5% of 49 in oral SCCs [85]. In our studies, aberrant cytoplasmic expression of p33^ING1b^, ING4, and ING5 was detected in 14.5%, 68.8%, and 47.7% in 214 cases of HNSCC, respectively [80, 86, 87], while no or seldom cytoplasmic expression of these ING proteins was detectable in the cases of normal mucosa. Nuclear localization of ING proteins is required for their normal function. Therefore, decreased nuclear expression of ING proteins, through either downregulation of nuclear expression or relocation from the nucleus to cytoplasm, may play a crucial role in the development and progression of HNSCC (Figure 3) and may be a new biomarker for the tumorigenesis of HNSCC.

The mechanism of translocation of ING proteins is not fully understood. Recently, a study from Riabowol's group demonstrated that p33^ING1b^ can especially bind members of the 14-3-3 family through phosphorylation at serine residue 199 [28]. 14-3-3 family members primarily reside in the cytoplasm and are associated with phosphorylated ligands involved in many cellular processes, including regulation of the cell cycle and DNA damage checkpoints [95]. 14-3-3 binding results in tethering of significant amounts of p33^ING1b^ in the cytoplasm [28]. Additionally, cytoplasmic p33^ING1b^ could be imported into the nucleus through interactions between its intrinsic NLS and karyopherins *α*2 and *β*1 [96]. In the nucleus, lamin A binds and targets ING1 and regulates ING1 levels and biological function [27]. Therefore, 14-3-3, karyopherins *α*2 and *β*1, and lamin A are involved in the dynamic regulation of subcellular distribution of ING1. Recently, we investigated the expression of p33^ING1b^ and 14-3-3*η* in 214 cases of HNSCC by immunohistochemistry and found that cytoplasmic p33^ING1b^ expression was significantly associated with 14-3-3*η* expression. Moreover, double immunofluorescence results confirmed the coexpression of p33^ING1b^ and 14-3-3*η* (unpublished data). These data indicated that 14-3-3*η* plays an important role in the cytoplasmic accumulation of p33^ING1b^ in HNSCC. However, the function of cytoplasmic ING is unclear and needs to be further studied.

There have been a few studies on the correlation between clinicopathological variables and expression of the *ING* genes. High LOH frequency of ING2 was statistically associated with advanced T stage, suggesting that ING2 LOH might occur at the late stage of HNSCC progression [82]. Although no clinicopathological variables were significantly related to the levels of ING3 mRNA, decreased expression of ING3 mRNA was associated with high mortality and was an independent prognostic factor for poor overall survival [92]. In our recent studies, no significant correlation was found between high nuclear expression of p33^ING1b^ and clinicopathological variables in HNSCC, but high expression of cytoplasmic p33^ING1b^ was significantly correlated with poor differentiation, T staging, lymph node metastasis, and TNM staging [86]. Also, high expression of nuclear ING4 in HNSCC was negatively correlated with poor differentiation, T staging, and TNM staging, while high expression of cytoplasmic ING4 in HNSCC was positively correlated with lymph node metastasis [93]. Also in the case of ING5, its nuclear expression correlated with differentiation of HNSCC, and abundant cytoplasmic expression correlated with poor differentiation [80].

## 11. Conclusions

 The* ING* family genes are supposed to belong to type II TSG and are involved in multiple cellular processes including chromatin remodeling, DNA repair, cell cycle control, senescence, and apoptosis. ING proteins are expressed independently of p53 status and function in both p53-dependent and p53-independent manner. Loss or downregulation of *ING* genes expression and/or translocation of ING proteins from the nucleus to the cytoplasm may play an important role in neoplastic development of HNSCC. Thus, the *ING* gene family could be a novel p53-independent biomarker for HNSCC. Further elucidation of the functions of ING family proteins, which can be either tumor suppressive or tumorigenic, will rationalize their application for a biomarker, and it will also reveal the potentiality of ING proteins as the therapeutic target [97].

## Figures and Tables

**Table 1:** *ING *gene mutation and expression in HNSCC.

*ING*	Origin	Methods	Mutation type/expression change	Position	Frequency	Reference
*ING1*	Patient	MM	LOH	13q34	20/44(45.5%)	[88]
	Cell lines	Sequencing	No mutation		0/5	
	Patient	Sequencing	No mutation		0/20	
	Patient	MM	LOH	13q33-34	23/34(68%)	[89]
	Patient	PCR-SSCP	Missense	PHD (215)	1/23(4.3%)	
	Patient	PCR-SSCP	Missense	PHD (216)	1/23(4.3%)	
	Patient	PCR-SSCP	Missense	NLS (192)	1/23(4.3%)	
	Patient	RT-PCR	Downregulation		6/12(50%)	[90]
	Patient	IHC	Downregulation		37/49(76%)	[85]
	Cell lines	Sequencing	No mutation		0/3	[86]
	Patient	IHC	Downregulation		79/214(36.9%)	

*ING2*	Patient	MM	LOH	4q35.1	33/55(54.6%)	[82]

*ING3*	Patient	MM	LOH	7q31	22/46(48%)	[91]
	Patient	RT-PCR	Downregulation		20/40(50%)	
	Patient	PCR-SSCP	Missense	LZL(20)	1/49(2%)	
	Patient	RT-PCR	Downregulation		37/71(52.1%)	[92]
	Patient	RT-PCR	Upregulation		15/71(21%)	

*ING4*	Patient	MM	LOH	12p13	33/50(66%)	[87]
	Patient	Sequencing	No mutation		0/50	
	Patient	Q-PCR	Downregulation		38/50(76%)	
	Patient	Q-PCR	Upregulation		7/50(14%)	
	Cell lines	Sequencing	No mutation		0/3	[93]
	Patient	IHC	Downregulation		96/214(44.9%)	

*ING5*	Patient	RT-PCR	Downregulation		19/31(61.3%)	[94]
	Patient	Sequencing	Missense	LZL(33)	1/31(3.2%)	
	Patient	Sequencing	Missense	NCR(68)	1/31(3.2%)	
	Patient	Sequencing	Missense	NCR(74)	1/31(3.2%)	
	Cell lines	Sequencing	No mutation		0/3	[80]
	Patient	IHC	Downregulation		77/214(36%)	

Note: MM, Microsatellite marker; PCR-SSCP, Polymerase chain reaction-single strand conformation polymorphism; RT-PCR, Retrotranscription-polymerase chain reaction; Q-PCR, Quantitative-polymerase chain reaction; IHC, immunohistochemistry.

## References

[1] Báez A (2008). Genetic and environmental factors in head and neck cancer genesis. *Journal of Environmental Science and Health C*.

[2] Gillison ML, Koch WM, Capone RB (2000). Evidence for a causal association between human papillomavirus and a subset of head and neck cancers. *Journal of the National Cancer Institute*.

[3] D’Souza G, Kreimer AR, Viscidi R (2007). Case-control study of human papillomavirus and oropharyngeal cancer. *The New England Journal of Medicine*.

[4] Haddad RI, Shin DM (2008). Recent advances in head and neck cancer. *The New England Journal of Medicine*.

[5] Lai S, Batsakis JG, Ordonez NG, Luna MA, Goepfert H, El-Naggar AK (1995). Genotypic and phenotypic alterations of p53 in head and neck squamous cell carcinoma. *Oncology Reports*.

[6] Gasco M, Crook T (2003). The p53 network in head and neck cancer. *Oral Oncology*.

[7] Weber A, Wittekind C, Tannapfel A (2003). Genetic and epigenetic alterations of 9p21 gene products in benign and malignant tumors of the head and neck. *Pathology Research and Practice*.

[8] Papadimitrakopoulou VA, Izzo J, Mao L (2001). Cyclin D1 and p16 alterations in advanced premalignant lesions of the upper aerodigestive tract: role in response to chemoprevention and cancer development. *Clinical Cancer Research*.

[9] Garkavtsev I, Kazarov A, Gudkov A, Riabowol K (1996). Suppression of the novel growth inhibitor p33(ING1) promotes neoplastic transformation. *Nature Genetics*.

[10] Nagashima M, Shiseki M, Miura K (2001). DNA damage-inducible gene p33ING2 negatively regulates cell proliferation through acetylation of p53. *Proceedings of the National Academy of Sciences of the United States of America*.

[11] Shimada Y, Saito A, Suzuki M, Takahashi E, Horie M (1998). Cloning of a novel gene (ING1L) homologous to ING1, a candidate tumor suppressor. *Cytogenetics and Cell Genetics*.

[12] Nagashima M, Shiseki M, Pedeux RM (2003). A novel PHD-finger motif protein, p47ING3, modulates p53-mediated transcription, cell cycle control, and apoptosis. *Oncogene*.

[13] Shiseki M, Nagashima M, Pedeux RM (2003). p29ING4 and p28ING5 bind to p53 and p300, and enhance p53 activity. *Cancer Research*.

[14] Ythier D, Larrieu D, Brambilla C, Brambilla E, Pedeux R (2008). The new tumor suppressor genes ING: genomic structure and status in cancer. *International Journal of Cancer*.

[15] Shah S, Smith H, Feng X, Rancourt DE, Riabowol K (2009). ING function in apoptosis in diverse model systems. *Biochemistry and Cell Biology*.

[16] He GHY, Helbing CC, Wagner MJ, Sensen CW, Riabowol K (2005). Phylogenetic analysis of the ING family of PHD finger proteins. *Molecular Biology and Evolution*.

[17] Saito A, Furukawa T, Fukushige S (2000). p24/ING1-ALT1 and p47/ING1-ALT2, distinct alternative transcripts of p33/ING1. *Journal of Human Genetics*.

[18] Unoki M, Kumamoto K, Robles AI, Shen JC, Zheng Z-M, Harris CC (2008). A novel ING2 isoform, ING2b, synergizes with ING2a to prevent cell cycle arrest and apoptosis. *FEBS Letters*.

[19] Unoki M, Shen JC, Zheng Z-M, Harris CC (2006). Novel splice variants of ING4 and their possible roles in the regulation of cell growth and motility. *Journal of Biological Chemistry*.

[20] Raho G, Miranda C, Tamborini E, Pierotti MA, Greco A (2007). Detection of novel mRNA splice variants of human ING4 tumor suppressor gene. *Oncogene*.

[21] Coles AH, Jones SN (2009). The ING gene family in the regulation of cell growth and tumorigenesis. *Journal of Cellular Physiology*.

[22] Peña PV, Davrazou F, Shi X (2006). Molecular mechanism of histone H3K4me3 recognition by plant homeodomain of ING2. *Nature*.

[23] Ha S, Park S, Yun CH, Choi Y (2002). Characterization of nuclear localization signal in mouse ING1 homolog protein. *Biochemical and Biophysical Research Communications*.

[24] Scott M, Boisvert F-M, Vieyra D, Johnston RN, Bazett-Jones DP, Riabowol K (2001). UV induces nucleolar translocation of ING1 through two distinct nucleolar targeting sequences. *Nucleic Acids Research*.

[25] Soliman MA, Riabowol K (2007). After a decade of study-ING, a PHD for a versatile family of proteins. *Trends in Biochemical Sciences*.

[26] Wang J, Chin MY, Li G (2006). The novel tumor suppressor p33ING2 enhances nucleotide excision repair via inducement of histone H4 acetylation and chromatin relaxation. *Cancer Research*.

[27] Han X, Feng X, Rattner JB (2008). Tethering by lamin A stabilizes and targets the ING1 tumour suppressor. *Nature Cell Biology*.

[28] Gong W, Russell M, Suzuki K, Riabowol K (2006). Subcellular targeting of p33ING1b by phosphorylation-dependent 14-3-3 binding regulates p21WAF1 expression. *Molecular and Cellular Biology*.

[29] Unoki M, Kumamoto K, Takenoshita S, Harris CC (2009). Reviewing the current classification of inhibitor of growth family proteins. *Cancer Science*.

[30] Jenuwein T, Allis CD (2001). Translating the histone code. *Science*.

[31] Skowyra D, Zeremski M, Neznanov N (2001). Differential association of products of alternative transcripts of the candidate tumor suppressor ING1 with the mSin3/HDAC1 transcriptional corepressor complex. *Journal of Biological Chemistry*.

[32] Vieyra D, Loewith R, Scott M (2002). Human ING1 proteins differentially regulate histone acetylation. *Journal of Biological Chemistry*.

[33] Kuzmichev A, Zhang Y, Erdjument-Bromage H, Tempst P, Reinberg D (2002). Role of the Sin3-histone deacetylase complex in growth regulation by the candidate tumor suppressor p33ING1. *Molecular and Cellular Biology*.

[34] Pedeux R, Sengupta S, Shen JC (2005). ING2 regulates the onset of replicative senescence by induction of p300-dependent p53 acetylation. *Molecular and Cellular Biology*.

[35] Wang Y, Wang J, Li G (2006). Leucine zipper-like domain is required for tumor suppressor ING2-mediated nucleotide excision repair and apoptosis. *FEBS Letters*.

[36] Doyon Y, Cayrou C, Ullah M (2006). ING tumor suppressor proteins are critical regulators of chromatin acetylation required for genome expression and perpetuation. *Molecular Cell*.

[37] Doyon Y, Selleck W, Lane WS, Tan S, Côté J (2004). Structural and functional conservation of the NuA4 histone acetyltransferase complex from yeast to humans. *Molecular and Cellular Biology*.

[38] Shi X, Hong T, Walter KL (2006). ING2 PHD domain links histone H3 lysine 4 methylation to active gene repression. *Nature*.

[39] Peña PV, Hom RA, Hung T (2008). Histone H3K4me3 binding is required for the DNA repair and apoptotic activities of ING1 tumor suppressor. *Journal of Molecular Biology*.

[40] Palacios A, Garcia P, Padró D, López-Hernández E, Martín I, Blanco FJ (2006). Solution structure and NMR characterization of the binding to methylated histone tails of the plant homeodomain finger of the tumour suppressor ING4. *FEBS Letters*.

[41] Palacios A, Muñoz IG, Pantoja-Uceda D (2008). Molecular basis of histone H3K4me3 recognition by ING4. *Journal of Biological Chemistry*.

[42] Hung T, Binda O, Champagne KS (2009). ING4 mediates crosstalk between histone H3K4 trimethylation and H3 acetylation to attenuate cellular transformation. *Molecular Cell*.

[43] Vicencio JM, Galluzzi L, Tajeddine N (2008). Senescence, apoptosis or autophagy? When a damaged cell must decide its path—a mini-review. *Gerontology*.

[44] Gordon PMK, Soliman MA, Bose P, Trinh Q, Sensen CW, Riabowol K (2008). Interspecies data mining to predict novel ING-protein interactions in human. *BMC Genomics*.

[45] Cheung K.-J. JR, Mitchell D, Lin P, Li G (2001). The tumor suppressor candidate p33ING1 mediates repair of UV-damaged DNA. *Cancer Research*.

[46] Campos EI, Martinka M, Mitchell DL, Dai DL, Li G (2004). Mutations of the ING1 tumor suppressor gene detected in human melanoma abrogate nucleotide excision repair. *International Journal of Oncology*.

[47] Scott M, Bonnefin P, Vieyra D (2001). UV-induced binding of ING1 to PCNA regulates the induction of apoptosis. *Journal of Cell Science*.

[48] Cheung K-JJ, Bush JA, Jia W, Li G (2000). Expression of the novel tumour suppressor p33(ING1) is independent of p53. *British Journal of Cancer*.

[49] Campisi J, D’Adda Di Fagagna F (2007). Cellular senescence: when bad things happen to good cells. *Nature Reviews Molecular Cell Biology*.

[50] Garkavtsev I, Riabowol K (1997). Extension of the replicative life span of human diploid fibroblasts by inhibition of the p33(ING1) candidate tumor suppressor. *Molecular and Cellular Biology*.

[51] Goeman F, Thormeyer D, Abad M (2005). Growth inhibition by the tumor suppressor p33ING1 in immortalized and primary cells: involvement of two silencing domains and effect of Ras. *Molecular and Cellular Biology*.

[52] Vieyra D, Toyama T, Hara Y, Boland D, Johnston R, Riabowol K (2002). ING1 isoforms differentially affect apoptosis in a cell age-dependent manner. *Cancer Research*.

[53] Pedeux R, Sengupta S, Shen JC (2005). ING2 regulates the onset of replicative senescence by induction of p300-dependent p53 acetylation. *Molecular and Cellular Biology*.

[54] Ohgi T, Masaki T, Nakai S (2002). Expression of p33ING1 in hepatocellular carcinoma: relationships to tumour differentiation and cyclin E kinase activity. *Scandinavian Journal of Gastroenterology*.

[55] Takahashi M, Seki N, Ozaki T (2002). Identification of the p33ING1-regulated genes that include cyclin B1 and protooncogene DEK by using cDNA microarray in a mouse mammary epithelial cell line NMuMG. *Cancer Research*.

[56] Xie YF, Sheng W, Xiang J, Zhang H, Ye Z, Yang J (2009). Adenovirus-mediated ING4 expression suppresses pancreatic carcinoma cell growth via induction of cell-cycle alteration, apoptosis, and inhibition of tumor angiogenesis. *Cancer Biotherapy and Radiopharmaceuticals*.

[57] Helbing GC, Veillette C, Riabowol K, Johnston RN, Garkavtsev I (1997). A novel candidate tumor suppressor, ING1, is involved in the regulation of apoptosis. *Cancer Research*.

[58] Garkavtsev I, Grigorian IA, Ossovskaya VS, Chernov MV, Chumakov PM, Gudkov AV (1998). The candidate tumour suppressor p33(ING1) cooperates with p53 in cell growth control. *Nature*.

[59] Shinoura N, Muramatsu Y, Nishimura M (1999). Adenovirus-mediated transfer of p33(ING1) with p53 drastically augments apoptosis in gliomas. *Cancer Research*.

[60] Feng X, Bonni S, Riabowol K (2006). HSP70 induction by ING proteins sensitizes cells to tumor necrosis factor alpha receptor-mediated apoptosis. *Molecular and Cellular Biology*.

[61] Gozani O, Karuman P, Jones DR (2003). The PHD finger of the chromatin-associated protein ING2 functions as a nuclear phosphoinositide receptor. *Cell*.

[62] Chin MY, Ng KCP, Li G (2005). The novel tumor suppressor p33ING2 enhances UVB-induced apoptosis in human melanoma cells. *Experimental Cell Research*.

[63] Wang Y, Li G (2006). ING3 promotes UV-induced apoptosis via Fas/caspase-8 pathway in melanoma cells. *Journal of Biological Chemistry*.

[64] Harris SL, Levine AJ (2005). The p53 pathway: positive and negative feedback loops. *Oncogene*.

[65] Berardi P, Russell M, El-Osta A, Riabowol K (2004). Functional links between transcription, DNA repair and apoptosis. *Cellular and Molecular Life Sciences*.

[66] Campos EI, Chin MY, Kuo WH, Li G (2004). Biological functions of the ING family tumor suppressors. *Cellular and Molecular Life Sciences*.

[67] Leung KM, Po LS, Tsang FC (2002). The candidate tumor suppressor ING1b can stabilize p53 by disrupting the regulation of p53 by MDM2. *Cancer Research*.

[68] Kichina JV, Zeremski M, Aris L (2006). Targeted disruption of the mouse ing1 locus results in reduced body size, hypersensitivity to radiation and elevated incidence of lymphomas. *Oncogene*.

[69] Coles AH, Liang H, Zhu Z (2007). Deletion of p37Ing1 in mice reveals a p53-independent role for Ing1 in the suppression of cell proliferation, apoptosis, and tumorigenesis. *Cancer Research*.

[70] Oki E, Maehara Y, Tokunaga E, Kakeji Y, Sugimachi K (1999). Reduced expression of p33(ING1) and the relationship with p53 expression in human gastric cancer. *Cancer Letters*.

[71] Kameyama K, Huang C-L, Liu D (2003). Reduced ING1b gene expression plays an important role in carcinogenesis of non-small cell lung cancer patients. *Clinical Cancer Research*.

[72] Garkavtsev I, Kozin SV, Chernova O (2004). The candidate tumour suppressor protein ING4 regulates brain tumour growth and angiogenesis. *Nature*.

[73] Nozell S, Laver T, Moseley D (2008). The ING4 tumor suppressor attenuates NF-*κ*B activity at the promoters of target genes. *Molecular and Cellular Biology*.

[74] Nouman GS, Angus B, Lunec J, Crosier S, Lodge A, Anderson JJ (2002). Comparative assessment expression of the inhibitor of growth 1 gene (ING1) in normal and neoplastic tissues. *Hybridoma and Hybridomics*.

[75] Nouman GS, Anderson JJ, Crosier S, Shrimankar J, Lunec J, Angus B (2003). Downregulation of nuclear expression of the p33ING1b inhibitor of growth protein in invasive carcinoma of the breast. *Journal of Clinical Pathology*.

[76] Li M, Jin Y, Sun W-J (2009). Reduced expression and novel splice variants of ING4 in human gastric adenocarcinoma. *Journal of Pathology*.

[77] Chen L, Matsubara N, Yoshino T (2001). Genetic alterations of candidate tumor suppressor ING1 in human esophageal squamous cell cancer. *Cancer Research*.

[78] Nouman GS, Anderson JJ, Wood KM (2002). Loss of nuclear expression of the p33ING1b inhibitor of growth protein in childhood acute lymphoblastic leukaemia. *Journal of Clinical Pathology*.

[79] Vieyra D, Senger DL, Toyam T (2003). Altered subcellular localization and low frequency of mutations of ING1 in human brain tumors. *Clinical Cancer Research*.

[80] Li X, Nishida T, Noguchi A (2010). Decreased nuclear expression and increased cytoplasmic expression of ING5 may be linked to tumorigenesis and progression in human head and neck squamous cell carcinoma. *Journal of Cancer Research and Clinical Oncology*.

[81] Lu F, Dai DL, Martinka M, Ho V, Li G (2006). Nuclear ING2 expression is reduced in human cutaneous melanomas. *British Journal of Cancer*.

[82] Borkosky SS, Gunduz M, Nagatsuka H (2009). Frequent deletion of ING2 locus at 4q35.1 associates with advanced tumor stage in head and neck squamous cell carcinoma. *Journal of Cancer Research and Clinical Oncology*.

[83] Kumamoto K, Fujita K, Kurotani R (2009). ING2 is upregulated in colon cancer and increases invasion by enhanced MMP13 expression. *International Journal of Cancer*.

[84] Sarker KP, Kataoka H, Chan A (2008). ING2 as a novel mediator of transforming growth factor-*β*-dependent responses in epithelial cells. *Journal of Biological Chemistry*.

[85] Zhang J-T, Wang D-W, Li Q-X (2008). Nuclear to cytoplasmic shift of p33ING1b protein from normal oral mucosa to oral squamous cell carcinoma in relation to clinicopathological variables. *Journal of Cancer Research and Clinical Oncology*.

[86] Li XH, Noguchi A, Nishida T Cytoplasmic expression of p33ING1b is correlated with tumorigenesis and progression of head and neck squamous cell carcinoma.

[87] Gunduz M, Nagatsuka H, Demircan K (2005). Frequent deletion and down-regulation of ING4, a candidate tumor suppressor gene at 12p13, in head and neck squamous cell carcinomas. *Gene*.

[88] Sanchez-Cespedes M, Okami K, Cairns P, Sidransky D (2000). Molecular analysis of the candidate tumor suppressor gene INGI in human head and neck tumors with 13q deletions. *Genes Chromosomes and Cancer*.

[89] Gunduz M, Ouchida M, Fukushima K (2000). Genomic structure of the human ING1 gene and tumor-specific mutations detected in head and neck squamous cell carcinomas. *Cancer Research*.

[90] Tachibana M, Shinagawa Y, Kawamata H (2003). RT-PCR amplification of RNA extracted from formalin-fixed, paraffin-embedded oral cancer sections: analysis of p53 pathway. *Anticancer Research*.

[91] Gunduz M, Ouchida M, Fukushima K (2002). Allelic loss and reduced expression of the ING3, a candidate tumor suppressor gene at 7q31, in human head and neck cancers. *Oncogene*.

[92] Gunduz M, Beder LB, Gunduz E (2008). Downregulation of ING3 mRNA expression predicts poor prognosis in head and neck cancer. *Cancer Science*.

[93] Li XH, Nishida T, Noguchi A Decreased nuclear expression and increased cytoplasmic expression of ING4 is correlated with tumorigenesis and progression of head and neck squamous cell carcinoma (HNSCC).

[94] Cengiz B, Gunduz E, Gunduz M (2010). Tumor-specific mutation and downregulation of ING5 detected in oral squamous cell carcinoma. *International Journal of Cancer*.

[95] Hermeking H, Benzinger A (2006). 14-3-3 proteins in cell cycle regulation. *Seminars in Cancer Biology*.

[96] Russell MW, Soliman MA, Schriemer D, Riabowol K (2008). ING1 protein targeting to the nucleus by karyopherins is necessary for activation of p21. *Biochemical and Biophysical Research Communications*.

[97] Unoki M, Kumamoto K, Harris CC (2009). ING proteins as potential anticancer drug targets. *Current Drug Targets*.

